# Decreased expression of a phagocytic receptor Siglec-1 on alveolar macrophages in chronic obstructive pulmonary disease

**DOI:** 10.1186/s12931-020-1297-2

**Published:** 2020-01-28

**Authors:** Atsushi Tanno, Naoya Fujino, Mitsuhiro Yamada, Hisatoshi Sugiura, Taizou Hirano, Rie Tanaka, Hirohito Sano, Satoshi Suzuki, Yoshinori Okada, Masakazu Ichinose

**Affiliations:** 1 Department of Respiratory Medicine, Tohoku University Graduate School of Medicine, 1-1 Seiryocho, Aobaku, Sendai, 980 8574 Japan; 2 Department of Thoracic Surgery, Japanese Red Cross Ishinomaki Hospital, Ishinomaki, 986 8522 Japan; 3 Department of Thoracic Surgery, Institute of Development, Aging and Cancer, Tohoku University, Sendai, 980 8575 Japan

**Keywords:** Chronic obstructive pulmonary disease, Alveolar macrophage, Phagocytosis, Siglec-1

## Abstract

**Background:**

Alveolar macrophages are professional phagocytes that remove microbial pathogens inhaled into the lung. The phagocytic ability is compromised in chronic obstructive pulmonary disease (COPD). However, the molecular mechanisms underlying this defect in phagocytosis are not clearly defined.

**Materials and methods:**

Cell suspensions were collected from lung tissues of patients undergoing lung resection. Alveolar macrophages were detected as FSC^hi^/ SSC^hi^/CD45^+^/CD206^+^ cells in the isolated cell suspension by flow-cytometry. The cell surface expression of plasma membrane-bound phagocytic receptors (Fcγ receptor I (FcγRI), a complement receptor CD11b, macrophage scavenger receptor-1 (MSR-1), CD36 and Siglec-1) was determined on the alveolar macrophages. Correlations between the expression levels of the phagocytic receptors and disease severity were analysed. Phagocytosis of fluorescence-tagged bacteria by human alveolar macrophages was evaluated.

**Results:**

The flow-cytometry analyses revealed that FcγRI, CD11b, MSR-1 and Siglec-1, but not CD36, were expressed on human alveolar macrophages. Among these receptors, Siglec-1 expression was significantly decreased on alveolar macrophages in COPD ex-smokers (*n* = 11), compared to control never-smokers (n = 11) or control ex-smokers (*n* = 9). The Siglec-1 expression on alveolar macrophages was significantly correlated with lung function (forced expiratory volume in 1 s) and with the severity of emphysema. Treatment of human alveolar macrophages with an anti-Siglec1 blocking antibody decreased phagocytosis of non-typeable *Haemophilus influenzae* (NTHi).

**Conclusion:**

Our findings demonstrated reduced expression of Siglec-1 on alveolar macrophages in COPD, which is involved in engulfment of NTHi.

## Background

Alveolar macrophages serve as professional phagocytes that have the ability to eliminate inhaled microorganisms or noxious particles and endogenous apoptotic cells or cellular debris for innate host defence and the resolution of inflammation [1]. However, in the lung of patients with chronic obstructive pulmonary disease (COPD), alveolar macrophages participate in pathological processes of chronic inflammation and tissue remodelling (i.e. alveolar destruction and small airway fibrosis) by producing reactive oxygen species (ROS), pro-inflammatory cytokines/chemokines, elastolytic proteases and pro-fibrotic mediators [2, 3]. In addition to the dysregulated production of the mediators, recent studies have indicated that alveolar macrophages of COPD were functionally defective in the phagocytosis of microbial pathogens, such as *Haemophilus influenzae* [4–6], *Streptococcus pneumoniae* [5] and *Moraxella catarrhalis* [6]. This defective phagocytosis of microbes is implicated in the bacterial colonisation and persistent inflammation in the airway of COPD, which are postulated to cause acute exacerbations [7].

Phagocytosis is defined as the cellular uptake of particles (> 0.5 μm) by a plasma-membrane envelope and consists of multiple biological processes: recognition, engulfment, plasma-membrane fusion, maturation of phagosomes with progressive acidification and culminating in phagolysosomal fusion and digestion [8]. Plasma membrane-receptors have a critical role in the sensing and engulfment of microbes in the initial phase of phagocytosis and are classified into (i) opsonic receptors (Fc receptors and complement receptors) and (ii) non-opsonic, pattern-recognition receptors (scavenger receptors and lectin-like recognition molecules) [9]. Scavenger receptors such as macrophage scavenger receptor-1 (MSR-1, also known as SR-A1), macrophage receptor with collagenous structure (MARCO, also known as SR-A2) and CD36 vary in the structure of their extracellular domains and recognise a large variety of molecules including not only microbial ligands (lipopolysaccharide and lipoteichoic acid) but also endogenous self-ligands (lipoproteins, unmodified proteins, etc.) [10]. Lectin-like recognition molecules also consist of diverse membrane-bound receptors such as C-type lectin receptors (mannose receptor or dectin-1, etc.) and sialic acid-binding immunoglobulin-like lectins (e.g. Siglec-1) [11]. This extremely diverse array of plasma membrane-receptors allows professional phagocytes to sense and eat a wide variety of foreign pathogens.

It is known that the expression of cell surface molecules is altered in alveolar macrophages of smokers or patients with COPD. Smoking reduces the expression of cell surface molecules involved in apoptotic cell-clearance (CD91) and cellular adhesion (CD31, CD44) [12]. Moreover molecules involved in antigen presentation (CD86 and CD11a) are down-regulated in the alveolar macrophages of COPD [13]. Despite the increasing evidence of the phenotypic alteration of alveolar macrophages under a pathological condition, the expression levels of receptors mediating bacterial phagocytosis have not been clearly determined.

In this study, we aimed to determine alteration of expression levels of phagocytosis-associated receptors in COPD and its functional consequences in bacterial uptake. Here we analysed representative membrane-bound receptors that are known to be involved in bacteria phagocytosis including Fcγ receptor I (FcγRI), CD11b (a subset of a complement receptor), MSR-1, CD36 and Siglec-1 (also knonwn as CD169). We directly analysed the cell surface expression of these receptors on alveolar macrophages freshly isolated from human lung tissues using flow-cytometry and found that Siglec-1 expression was significantly decreased in alveolar macrophages isolated from lung tissues of patients with COPD. Furthermore we confirmed that a blocking antibody for Siglec-1 reduced the phagocytosis capability of non-typeable *Haemophilus influenzae* in human alveolar macrophages.

## Methods

### Patient population

Subjects for the flow-cytometric analysis and immunostaining of phagocytic receptor expression participated in the study from September 2016 to August 2017. Subjects for the phagocytosis study participated between January 2018 to August 2019. All patients with COPD satisfied the Global Initiative for Chronic Obstructive Lung Disease (GOLD) guideline criteria [14]. Control ex-smokers and COPD ex-smokers had ceased smoking for more than 2 months before the enrolment. We excluded current smokers and subjects with asthma, bronchiectasis, pulmonary fibrosis or other inflammatory lung diseases. Written informed consent was obtained from all subjects who participated in this study. This study was approved by the ethics committees at Tohoku University School Graduate of Medicine (registration number: 2017–1-352) and Japanese Red Cross Ishinomaki Hospital (registration number: 13–12).

### Chest CT assessment of low attenuation areas

We evaluated low attenuation area scores using a high-resolution CT scan as an indicator for pulmonary emphysema according to the Goddard classification [15]. Briefly low attenuation value was scored for the bilateral upper, middle and lower lung fields. Zero represented no emphysema; 1 was given for up to 25%, 2 for up to 50%, 3 for up to 75% and 4 for total involvement or almost total absence of normal lung tissues. Scores ranged from 0 to 24.

### Preparation of lung cell suspension

Lung tissues were provided by patients who underwent lung resection due to primary lung cancer. We verified that the tissues had no pathological findings of tumours or overt fibrosis. Resected lung tissues were immediately immersed in StemSurvive-Lung tissue preservation solution (Kurabo Industries Ltd., Osaka, Japan), stored at 4 °C and used within 12 hours after surgery.

### Flow-cytometry analysis, fluorescence-activated cell sorting and diff-Quik staining

We collected lung hematopoietic cells including alveolar macrophages by perfusing and lavaging tissues with saline. Detailed methods for flow-cytometric analyses were described in the online supplement.

### Preparation of heat-killed and fluorescent dye-tagged non-typeable *Haemophilus influenzae*

Non-typeable *Haemophilus influenzae* (NTHi; GTC 15013, JNBP_02694) was obtained from Gifu University Center for Conservation of Microbial Genetic Resource. The number of bacteria was counted with Bacteria Counting Kit (Thermo Fisher Scientific). NTHi was killed at 65 °C for 1 hour and was labeled with pHrode Phagocytosis Particle Labeling Kit for Flow Cytometry (Thermo Fisher Scientific) according to the manufacturer’s instruction.

### Phagocytosis assay

Lung tissue was perfused with normal saline. The retrieved cell suspension was centrifuged at 400 *g* for 30 min at 20 °C. Cellular fractions in the cell suspensions were routinely evaluated by Diff-Quik staining. The cell pellet was resuspended in PBS and subjected to Ficoll-Paque (GE Healthcare) density gradient centrifugation to remove granulocytes and erythrocytes. The mononuclear cell layer at the interface, mainly containing alveolar macrophages, was collected and washed with PBS. 6 × 10^5^ cells were reconstituted into 2 mL of HEPES-buffered RPMI 1640 (Thermo Fisher Scientific) containing 1% IgG/protease-free BSA. 3 × 10^4^ cells in 100 μL of the cell suspension were transferred into each well of a 96 well formatted plate and incubated for 2 hours in the 5% CO_2_ incubator at 37 °C. Anti-human Siglec-1 blocking antibody (BIO-RAD, clone 7–239, cat# MCA2517EL) or an isotype-matched control antibody (mouse IgG_1_; BIO-RAD, cat# MCA 928EL) was added and incubated for 1 hour at room temperature in order to block Siglec-1 on the surface of alveolar macrophages [16]. The final concentration of these antibodies was 10 μg/mL, which was sufficient to block binding of additional phycoerythrin-conjugated anti-human Siglec-1 antibody (eBioscience, clone 7–239, cat# 12–1699-42). This concentration of the antibody was not influential in cell viability (data not shown). Heat-killed and pHrode-labeled NTHi at 100 MOI was added onto the culture media. After 1 hour, cells were collected with 0.5% Trypsin/EDTA. Positive cells that engulfed pHrode-labeled bacteria in acidic phagosome compartments were quantified by flow-cytometry using FACS CantoII (BD Biosciences). Data were analysed by FlowJo (Tree Star Inc., OR, USA).

### Statistics

All data are expressed as medians (with interquartile ranges) unless otherwise indicated. Statistical tests were performed using GraphPad Prism 8 (GraphPad Software Inc., CA, USA). Difference in the distribution of the genders between CNS, CES and COPD were analysed using the Chi square test. For comparison between three groups, the Kruskal-Wallis test followed by the Dunn’s multiple comparison test was performed. Statistical correlation analyses were performed using Spearman’s test. Multivariable regression analyses were performed for evaluating the effect of potentially confounding factors. *P*-values less than 0.05 were considered significant.

## Results

### Characterisation of phagocytosis-associated receptor expression on alveolar macrophages isolated from lung tissues of patients with COPD

**Fig. 1:**
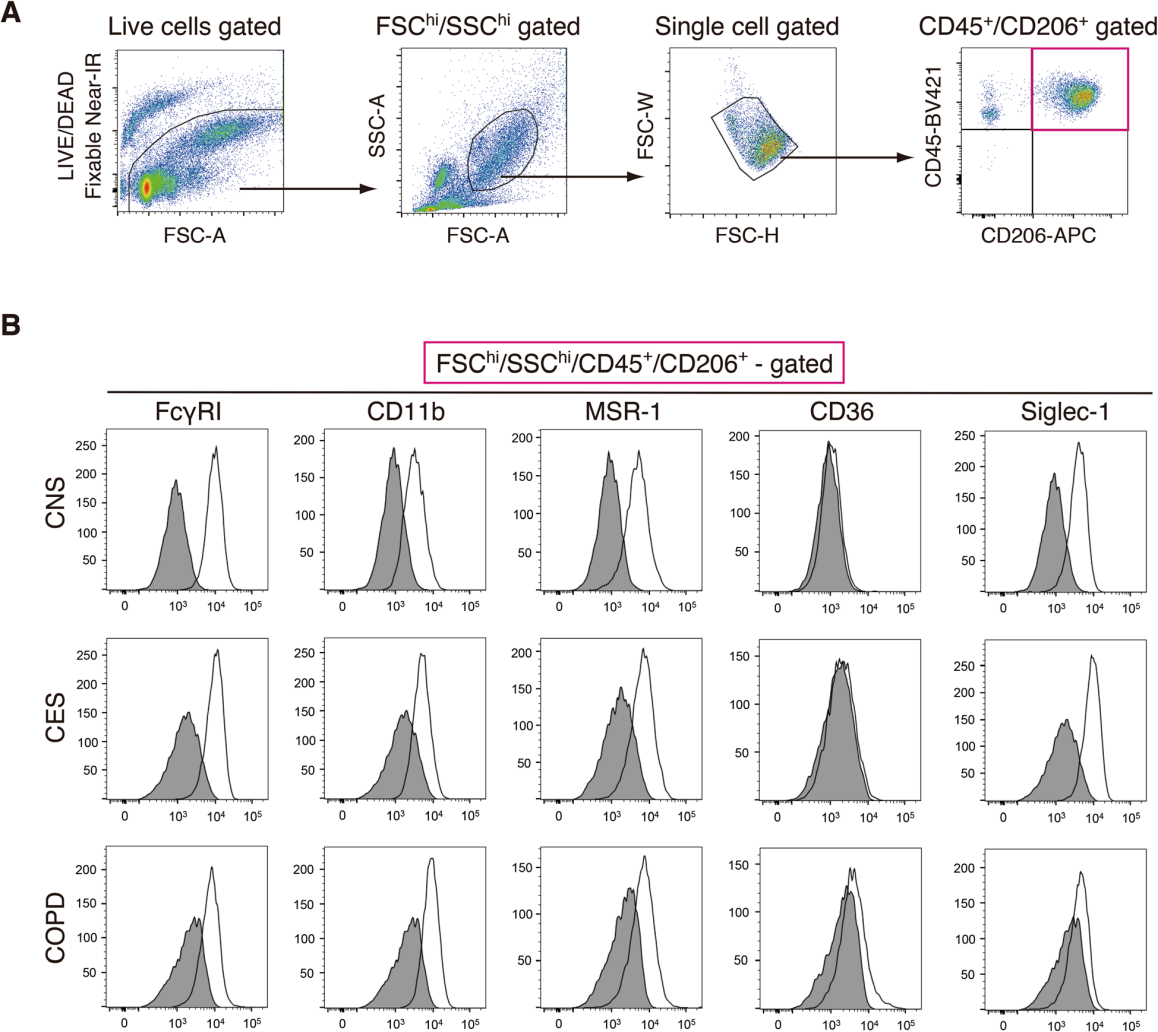
Expression levels of phagocytic receptors on human alveolar macrophages. **a** FACS gating strategy to delineate alveolar macrophages. Cell suspension was collected by perfusing and lavaging human lung tissues with saline. Alveolar macrophages were detected as live, FSC^hi^/SSC^hi^, single cell-gated CD45^+^/CD206^+^ cells in the cell suspension. **b** Representative histograms for the expression levels of FcγRI, CD11b, macrophage scavenger receptor-1 (MSR-1), CD36 and Siglec-1, on FSC^hi^/SSC^hi^/CD45^+^/CD206^+^ alveolar macrophages of control never-smokers (CNS, *n* = 11), control ex-smokers (CES, *n* = 9) and COPD ex-smokers (COPD, *n* = 11). Specific staining, open; isotype control, shaded

To examine cell surface expression levels of phagocytosis-associated receptors on human alveolar macrophages, we collected cell suspension by perfusing and lavaging peripheral lung tissues with saline and delineated alveolar macrophages as FSC^hi^/SSC^hi^/CD45^+^/CD206^+^ cells in flow-cytometry according to previous studies [2, 3] (Fig. 1a). We confirmed that FSC^hi^/SSC^hi^/CD45^+^/CD206^+^ cells from human lung tissue lavage were alveolar macrophages in three ways. First, cytospin samples of sorted FSC^hi^/SSC^hi^/CD45^+^/CD206^+^ cells exhibited homogenous, basophilic large cells that were morphologically consistent with alveolar macrophages (Additional file 1: Figure S1A). Second, immunohistochemistry verified that CD206 was expressed by alveolar macrophages in human lung sections and its expression pattern was not different between control never-smokers (CNS), control ex-smokers (CES) and COPD ex-smokers (COPD) (Additional file 1: Figure S1B). Third, FSC^hi^/SSC^hi^/CD45^+^/CD206^+^ cells expressed CD14 at a minimum level, which was consisting with the previous reports [2, 3] (Additional file 1: Figure S1C).

**Fig. 2:**
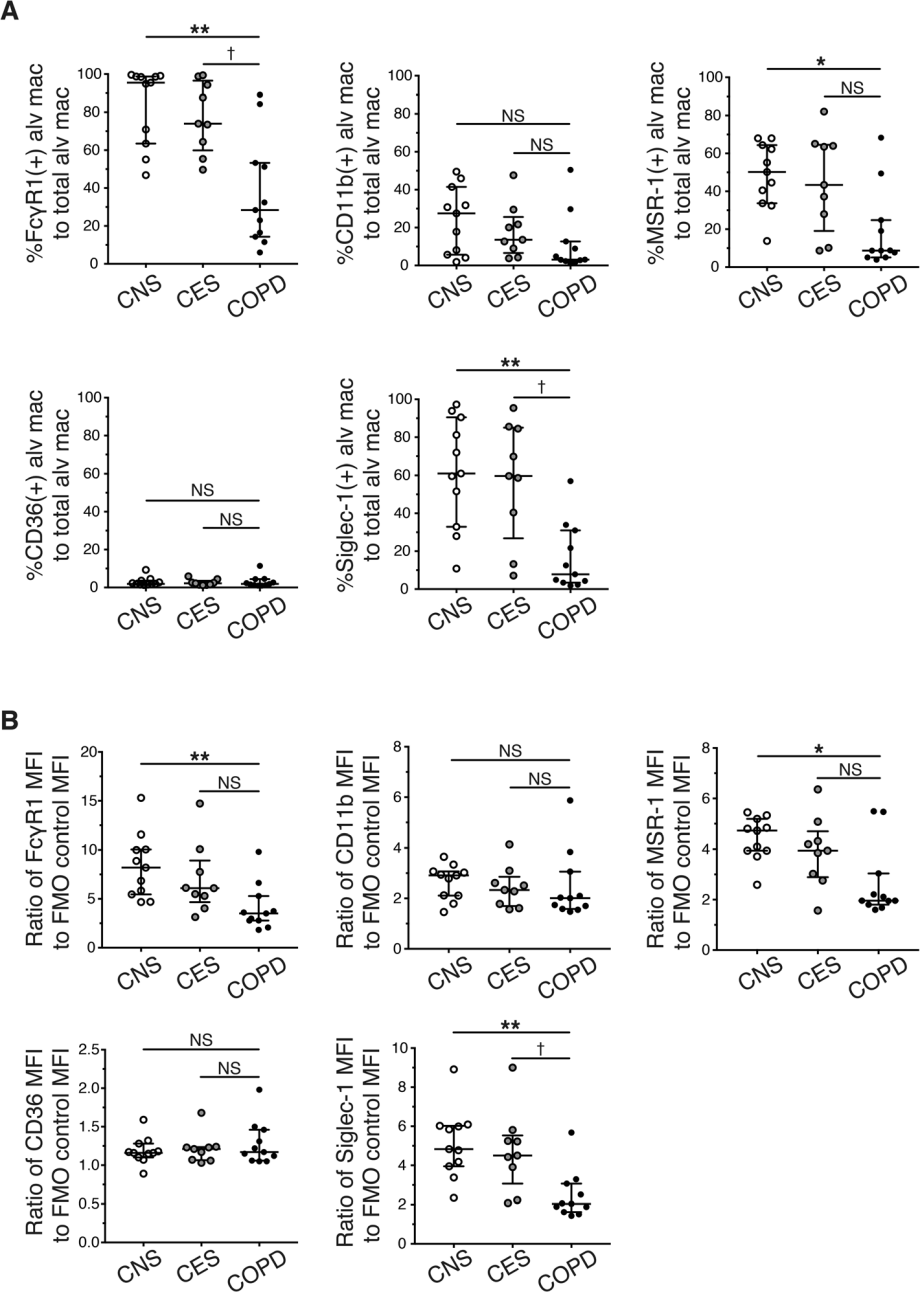
A decrease in cell surface expression of Siglec-1 on alveolar macrophages isolated from COPD lungs. Each antigen expression is shown as (**a**) the percentage of positive alveolar macrophages expressing each phagocytic receptor to the total alveolar macrophages and (**b**) the ratio of the geometric mean of the phagocytic receptor expression levels to the geometric mean of the corresponding isotype control in control never-smokers (CNS, n = 11, white circles), control ex-smokers (CES, *n* = 9 grey circles) and COPD ex-smokers (COPD, *n* = 11, black circles). Data represent median with interquartile ranges and were analysed with Kruskal-Wallis test followed by Dunn’s Multiple Comparison Test. *P < 0.05, **P < 0.01 compared with CNS; †P < 0.05 compared with CES. N.S. indicates “not significant”

We then examined the expression of each of phagocytosis-associated receptors (FcγRI, CD11b, MSR-1, CD36 and Siglec-1) on the surface of FSC^hi^/SSC^hi^/CD45^+^/CD206^+^ alveolar macrophages in flow-cytometry, that were collected from lung tissues of CNS (*n* = 11), CES (*n* = 9) and COPD with mild-to-moderate airflow limitation severity (*n* = 5 in GOLD stage1, *n* = 6 in GOLD stage2). The patient characteristics in the flow-cytometric analysis are shown in Table 1. We evaluated the receptor expression by two parameters: (i) the percentage of alveolar macrophages expressing each of the receptors to total alveolar macrophages and (ii) the geometric mean of the receptor expression intensity normalised by that of a corresponding fluorochrome minus one (FMO) control with an appropriate isotype control antibody. We found that FcγRI, CD11b, MSR-1 and Siglec-1 were expressed by alveolar macrophages isolated from CNS but CD36 exhibited no or little expression (Fig. 1b). The percentage of alveolar macrophages expressing FcγRI or Siglec-1 was significantly decreased in COPD subjects compared to CNS and CES (Fig. 2a). Moreover the percentage of alveolar macrophages expressing MSR-1 was slightly decreased in COPD subjects compared to CES, although its decrease was significant between COPD and CNS (Fig. 2a). Among these receptors, the reduction of Siglec-1 expression intensity on alveolar macrophages of COPD patients was statistically significant compared to CNS and CES (Fig. 2b). Although the difference in the expression intensity of FcγRI and MSR-1 of between CES and COPD was not statistically significant, the intensity of these receptors was decreased in COPD compared to CNS (Fig. 2b).

**Table 1:** Patient characteristics in flow-cytometry analyses and immunohistochemistry results of phagocytic receptor expression of alveolar macrophages

	Control never-smokers	Control ex-smokers	COPD ex-smokers
Subjects (n)	11	9	11
Men/women	1/10	7/2***	11/0****
Age	72 (64.0–77.0)	75 (65.5–77.5)	67 (64.0–73.0)
Smoking (pack-years)	0.0 (0.0–0.0)	33.0 (18.3–49.1)**	43.0 (30.0–68.8)****
Duration of smoking cessation (months)	NA	48.0 (2.0–168.0)	12.0 (2.0–120.0)
FVC (%pred)	100.7 (94.1–123.5)	95.2 (86.7–106.9)	112.0 (101.5–123.3)
FEV_1_ (%pred)	104.7 (98.6–119.4)	90.5 (83.0–94.7)	78.7 (66.8–93.4)***
FEV_1_/FVC (%)	80.7 (74.9–85.2)	76.3 (73.5–78.7)	62.6 (54.0–64.7)****††
LAA score	0 (0–0)	0 (0–0)	5 (4–9) ***†††
GOLD Grade (1/2/3/4)	NA	NA	5/6/0/0
Exacerbations per year	NA	NA	0
Treatment			
LABA	NA	NA	1
LAMA	NA	NA	5
LABA/LAMA	NA	NA	0
ICS	NA	NA	0
ICS/LABA	NA	NA	0
ICS/LABA/LAMA	NA	NA	0
Theophylline	NA	NA	0
Viable cell number per gram tissue^a^	7.4 × 10^6^ (4.9 × 10^6^–1.3 × 10^7^)	1.1 × 10^7^ (7.5 × 10^6^–2.3 × 10^7^)	1.1 × 10^7^ (8.8 × 10^6^–1.9 × 10^7^)
Viability (%)^b^	88 (83–91)	90 (85–92)	88 (85–92)

Data are presented as the median (with interquartile ranges). Data were analysed with Chi-square test, Kruskal-Wallis test followed by Dunn’s multiple comparison test or Mann-Whitney test

^a^Viable cell number per gram tissue, the number of whole viable cells that were collected from 1 gram of lung tissues

^b^Viability was evaluated with trypan blue staining

**P < 0.01, ***P < 0.001, ****P < 0.0001 compared with control never-smokers; ††*P* < 0.01, †††*P* < 0.001 compared with control ex-smokers

*FVC* forced vital capacity; *FEV*_*1*_, forced expiratory volume in 1 s; *FEV*_*1*_*%pred* % predicted values of FEV_1_; *LAA*, low attenuation area; *GOLD* Global Initiative for Chronic Obstructive Lung Disease; *LABA* long-acting β agonists; *LAMA* long-acting muscarinic antagonists; *ICS* inhaled corticosteroids; *NA* not applicable

### Relationship between phagocytosis-associated receptor expression on alveolar macrophages and clinical variables

Considering the differences in the expression level of FcγRI, MSR-1 and Siglec-1 on alveolar macrophages between CNS, CES and COPD, we next sought to determine whether these differences were associated with clinical variables such as age, gender, smoking history, airflow limitation severity and pulmonary emphysema. The severity of airflow limitation was evaluated as % predicted values of forced expiratory volume in 1 second (FEV_1_%pred). Pulmonary emphysema was quantified by measuring low attenuation areas on chest computed tomography (CT) images. We found that smoking history, FEV_1_%pred and low attenuation area scores exhibited a significant association with the phagocytosis-associated receptor expression (Table 2). A multiple linear regression analysis revealed that the severity of pulmonary emphysema was independently associated with the reduction of Siglec-1 expression on alveolar macrophages (Table 3).

**Table 2:** Correlation of clinical variables and phagocytosis-associated receptor expression on alveolar macrophages (univariable analysis)

		FcγRI		MSR-1		Siglec-1	
		MFI ratio	% positive	MFI	% positive	MFI	% positive
Age	*r*_*s*_	0.2735	0.1550	0.1842	0.1776	0.3462	0.3061
	*P* value	0.1365	0.4051	0.3213	0.3392	0.0564	0.0940
Gender	*r*_*s*_	−0.2666	−0.3258	− 0.1852	− 0.2889	−0.3184	−0.3184
	*P* value	0.0737	0.3185	0.1149	0.0809	0.0809	0.0737
Smoking history (Pack-years)	*r*_*s*_	−0.3670	− 0.3645	− 0.3568	− 0.3817	− 0.5333	− 0.4753
	*P* value	**0.0423**	**0.0438**	**0.0488**	**0.0341**	**0.0020**	**0.0069**
FEV_1_ (% pred)	*r*_*s*_	0.4140	0.3533	0.5115	0.4459	0.4656	0.3958
	*P* value	**0.0206**	0.0512	**0.0033**	**0.0119**	**0.0083**	**0.0275**
Low attenuation area scores	*r*_*s*_	−0.5358	−0.5577	−0.4564	−0.4945	−0.6778	−0.6440
	*P* value	**0.0019**	**0.0011**	**0.0099**	**0.0047**	**< 0.0001**	**< 0.0001**

Bold denotes values *P* < 0.05

*r*_*s*_, Spearman rank correlation coefficient. FEV_1_, forced expiratory volume in 1 second. MFI, mean fluorescence intensity

MFI ratio indicates the ratio of receptor MFI to the corresponding FMO control MFI

% positive indicates the percentage of a receptor-positive alveolar macrophages to total alveolar macrophages

**Table 3:** Correlation of clinical variables and phagocytosis-associated receptor expression of alveolar macrophages (multiple linear regression analysis)

		FcγRI		MSR-1		Siglec-1	
		MFI ratio	% positive	MFI	% positive	MFI	% positive
Smoking history (Pack-years)	Estimate	0.0116	−0.1288	0.0105	0.0514	−0.0193	−0.2747
	*P* value	0.7078	0.6213	0.2396	0.8057	0.2488	0.2935
FEV_1_ (% pred)	Estimate	0.0192	0.1153	0.0297	0.3519	−0.0057	−0.1107
	*P* value	0.7158	0.7946	0.0548	0.3265	0.8397	0.8016
Low attenuation area scores	Estimate	−0.4466	−3.8890	−0.0002	−2.6940	−0.2661	−5.0410
	*P* value	0.0731	0.0637	0.9982	0.1070	**0.0463**	**0.0180**

Estimate represents parameter estimate

Bold denotes values *P* < 0.05

FEV_1_, forced expiratory volume in one second. MFI, mean fluorescence intensity

MFI ratio indicates the ratio of receptor MFI to the corresponding FMO control MFI

% positive indicates the percentage of a receptor-positive alveolar macrophages to total alveolar macrophages

### Siglec-1 was important for engulfment of non-typeable *Haemophilus influenzae* by human alveolar macrophages

**Fig. 3:**
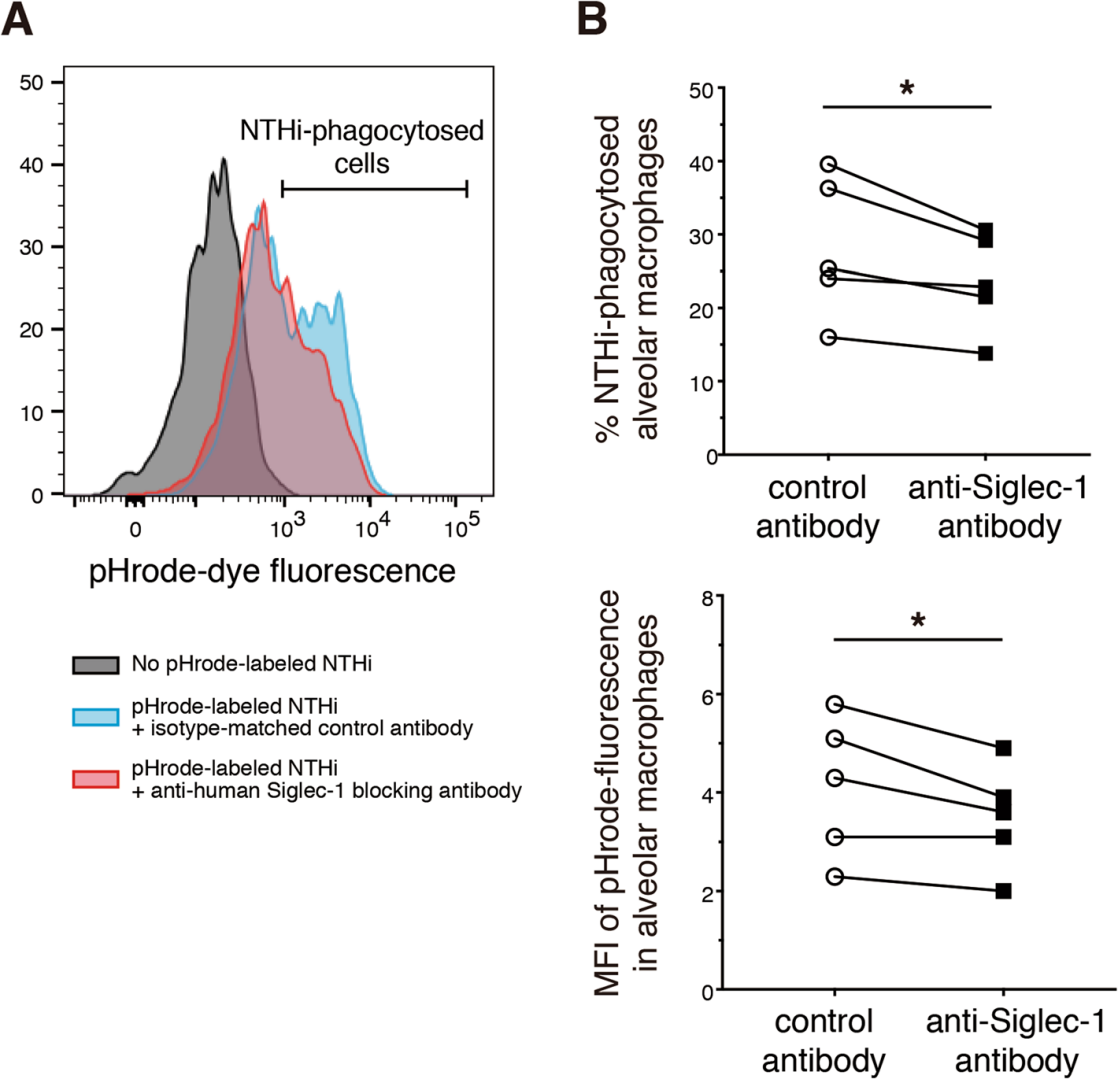
An anti-human Siglec-1 blocking antibody reduced engulfment of NTHi by human alveolar macrophages ex vivo. **a** Alveolar macrophages isolated from resected lung tissues of control never smokers were incubated with heat-killed and pHrode dye-labeled NTHi in the presence of an anti-human Siglec-1 blocking antibody or an isotype-matched control antibody. The bacterial engulfment was evaluated by flowcytometry. A representative histogram of pHrode-dye fluorescence was shown. A grey line indicated cells without pHrode-labeled NTHi as a negative control. A blue line showed cells treated with pHrode-labeled NTHi in the presence of an isotype-matched control antibody. A red line represented cells treated with pHrode-labeled NTHi in the presence of an anti-human Siglec-1 blocking antibody. **b** Bacterial engulfment was quantified by the percentage of NTHi-phagocytosed alveolar macrophages (upper panel) or by the mean fluorescence intensity (MFI) of pHrode-fluorescence in alveolar macrophages (lower panel). Anti-Siglec-1 antibody treatment reduced the engulfment ability (*N* = 5, *P < 0.05, Paired t-test)

So far we observed the significant reduction of Siglec-1 protein expression on alveolar macrophages of patients with COPD. Siglec-1 is known to recognise sialylated bacteria such as *Neisseria meningitidis* [17]. Considering that non-typeable *Haemophilus influenzae* (NTHi) was a sialylated pathogen and was mainly associated with exacerbation and bacterial colonisation of COPD, we therefore sought to determine whether Siglec-1 was functionally important for uptake of NTHi by human alveolar macrophages. As we wished to evaluate how Siglec-1 affected engulfment at the initial stage of bacterial phagocytosis, but not microbicidal efficacy in the late stage, we used heat-killed, fluorescent dye-tagged NTHi that increased in fluorescence under an acidic condition in phagolysosomes. We evaluated alveolar macrophages isolated from control never smokers that expressed Siglec-1 as shown by immunohistochemistry (Additional file 1: Figure S2). The patients’ characteristics was shown in Table 4. Cellular fractions of collected cell suspensions were as follows: macrophages, 89.0 ± 2.3%; lymphocytes, 6.2 ± 2.0%; neutrophils, 3.8 ± 1.5%; eosinophils, 0.4 ± 0.3%; monocytes, 0.8 ± 0.3%. After density gradient centrifugation to remove granulocytes, mononuclear cells were seeded on culture plates, most of which were thought to be alveolar macrophages. Then, adherent cells were cultured in the presence of an anti-Siglec-1 blocking antibody to inhibit biding of bacteria to Siglec-1 or an isotype-matched control antibody, which was followed by incubation with fluorescent dye-labeled NTHi. A flow-cytometry analysis indicated that the anti-Siglec-1 blocking antibody significantly reduced the percentage of NTHi-engulfed macrophages or the mean fluorescence intensity in macrophages (Fig. 3).

**Table 4:** Patient characteristics in the phagocytosis assay

	Control never-smokers
Subjects (n)	5
Men/women	0/5
Age	72.5 (65.8–74.8)
Smoking (pack-years)	0.0 (0.0–0.0)
FVC (%pred)	114.7 (107.4–122.6)
FEV_1_ (%pred)	116.6 (113.1–119.8)
FEV_1_/FVC (%)	80.9 (75.7–83.0)
Viable cell number per gram tissue^a^	3.1 × 10^6^ (2.9 × 10^6^–3.8 × 106)
Viability (%)^b^	88 (77–90)

Data are presented as the median (with interquartile ranges)

^a^Viable cell number per gram tissue, the number of whole viable cells that were collected from one gram of lung tissues

^b^Viability was evaluated with trypan blue staining

*FVC* forced vital capacity; *FEV*_*1*_ forced expiratory volume in 1 s; *FEV*_*1*_*%pred* % predicted values of FEV_1_

## Discussion

Our data suggested two novel aspects for human alveolar macrophages which may explain the compromised phagocytic ability of the alveolar macrophages in COPD; (i) the extracellular expression of plasma membrane-bound receptor for phagocytosis, Siglec-1, could be altered under pathological conditions in COPD lung and (ii) Siglec-1 was at least partially involved in the engulfment of NTHi by human alveolar macrophages. Importantly we observed reduced Siglec-1 expression in mild-to-moderate COPD subjects who had never experienced exacerbations, indicating that phagocytic receptor expression could be altered in early stages of COPD.

Siglecs (sialic acid-binding immunoglobulin (Ig)-like lectins) are type 1 membrane proteins containing a homologous N-terminal V-set Ig-like domain that recognises sialylated glycan, followed by variable numbers of C2 set domains [18]. The Siglec family is classified into two subfamilies: (i) Siglecs that are conserved across mammals (Siglec-1, CD22, myelin-associated glycoprotein and Siglec-15) and (ii) CD33-related Siglecs that are variable across mammals and therefore are postulated to have evolved rapidly [17]. Most Siglecs except for Siglec-1 have cytoplasmic regions that mediate intracellular signalling and regulate innate and adaptive immunity. CD22 and most CD33-related Siglecs have immunoreceptor tyrosine-based inhibitory motifs (ITIMs) in the cytoplasmic domains [18], that suppress activation signals by recruiting cytoplasmic phosphatases containing a Src homology 2 (SH2) domain [19]. Conversely a few Siglecs, such as Siglec-14, coupled with immunoreceptor tyrosine-based activating motifs (ITAMs)-bearing adaptors have the ability to activate immune cells via spleen tyrosine kinase (Syk) [17].

Some emerging evidence from human genetic studies has suggested that Siglecs are involved in pathogenesis of COPD. Null allele of *SIGLEC14* encoding immune-activating Siglec-14 was associated with a reduced risk of COPD exacerbation, possibly via suppression of pro-inflammatory responses by inactivation of Syk [20]. An integrative genomics approach utilising single nucleotide polymorphisms (SNPs) on COPD phenotypes and a genome-wide expression quantitative trait loci (eQTLs) study on lung tissues revealed that CD22 was one of potential causal genes for airflow limitation in patients with COPD [21]. However, to our knowledge, there has been no report showing a relationship between Siglec-1 and COPD.

Siglec-1 (also known as sialoadhesin or CD169) preferentially binds to α2,3-linked sialic acids [22, 23] and has longer extracellular regions (i.e., 17 Ig domains) that lack intracellular signalling motifs [24]. This unique structure of Siglec-1 may enable extension of sialic acid-binding sites outside the plasma membranes [25] and probably is useful for recognition, capture and uptake of microbes [17]. Siglec-1 is expressed by tissue macrophages and has a critical role in recognition and clearance of sialylated bacterial pathogens such as *Neisseria meningitidis* [26], *Campyrobacter jejuni* [27] and group B streptococcus [28]. In addition, Siglec-1 was required for proper pro-inflammatory responses via tumour necrosis factor-α and interferon (IFN)-β to eliminate *C.jejuni* that was intravenously administered to mice [29]. NTHi is also known to be a sialylated microbe and has genes encoding α2,3-sialyltransferase which adds sialic acids onto its lipooligosaccharide [30, 31]. Considering a number of evidence that NTHi is one of the most common bacteria involved in bacterial colonisation and acute exacerbation of COPD [32, 33], our data suggest that the reduced capability of alveolar macrophage phagocytosis for NTHi in COPD is due to the decrease in the extracellular expression of Siglec-1.

Although accumulating studies observed the defective phagocytosis of microbial pathogens including NTHi and *S.pneumoniae* by alveolar macrophages in COPD [4–6], there are few reports that identified molecular mechanisms involved in the reduced phagocytic ability. Bewley et al. recently reported that up-regulation of the anti-apoptotic protein Mcl-1 and a failure to produce mitochondrial ROS in alveolar macrophages of COPD reduced intracellular killing of *S.pneumoniae* [34]. In addition Harvey et al. reported that Nrf2 activation improved the phagocytic ability for NTHi by increasing the scavenger receptor MARCO in alveolar macrophages derived from patients with COPD, though it has not been examined whether MARCO protein expression is down-regulated on the surface of alveolar macrophages of COPD compared to control smokers [35]. Thus our study firstly showed the possibility that an alteration of plasma membrane-bound phagocytic receptors might be a critical indicator for bacterial clearance of alveolar macrophages under inflammatory conditions.

The multivariable linear regression analysis revealed that the severity of pulmonary emphysema evaluated by the low attenuation area score on chest CT scans was an independent parameter associated with the decrease in Siglec-1 on alveolar macrophages. Although type 1 interferon was known to induce Siglec-1 protein expression in patients with systemic sclerosis [36] or with human immunodeficiency virus-1 [37], it has not been determined whether and how the Siglec-1 protein expression is down-regulated during pathological processes. Thus our finding provides new evidence of the reduced expression of Siglec-1 in COPD of which pathogenesis is involved in chronic inflammation related to excessive oxidative stress and would provide a rationale to investigate molecular basis of upstream signalling pathways negatively regulating Siglec-1 expression.

A large number of studies support the idea that Siglec-1-expressing tissue macrophages have critical roles in initiation of proper pro-inflammatory responses to maintain homeostasis in viral infection, cancer and autoimmune diseases [11, 17, 38]. This idea was further validated by specific depletion of Siglec-1-positive macrophages using Siglec-1-diphteria toxin receptor-transgenic mice [39, 40]. Taken together with these reports, our observation suggests that Siglec-1^dim-neg^ alveolar macrophages in COPD lungs might represent a dysfunctional macrophage for innate host defence.

There are some limitations in our study. First, the sample size of our flow-cytometric analysis was small with only mild and moderate COPD, because the availability of resected lung tissues, especially from patients with severe or very severe COPD, was limited. However it is noteworthy that Siglec-1 was decreased even in the early stages of COPD patients who had never experienced acute exacerbation. Second, we did not investigate a prospective cohort to determine whether patients with COPD who have lower expression of Siglec-1 on their alveolar macrophages exhibited higher bacterial colonisation or experienced more acute exacerbations. Further functional studies with prospective larger cohorts are needed to precisely define the roles of Siglec-1 on alveolar macrophages in innate host defence under pathologic conditions. Third, as we performed the in vitro phagocytosis assay with female control never smokers, we cannot exclude a possibility that gender and smoking status might affect this result. Fourth, because all patients in this study had primary lung cancers, the presence of tumour cells might suppress the expressions of phagocytic receptors and the ability of bacterial phagocytosis. Although we used peripheral lung tissues distant from tumor lesions, recent reports have indicated that cancer cells might have a crosstalk with distant cells via extracellular vesicles to inhibit anti-tumour immunity [41]. Fifth, small numbers of dendritic cells and monocytes might be included in the phagocytosis assay, since we did not exclude those cells using specific cell surface markers in flow cytometry. Finally, although we observed decreases in expression of FcγRI and MSR-1 in COPD macrophages compared to CNS, we did not determine whether Siglec-1^dim-neg^ macrophages also lost expression of FcγRI and MSR-1 or if decreased receptor expression in COPD alveolar macrophages had less specificity. It would be notable for a future study to evaluate multiple receptor expression of alveolar macrophages in diseased lungs.

## Conclusions

We demonstrated that the extracellular expression of Siglec-1 was decreased on alveolar macrophages of COPD especially with pulmonary emphysema. Considering the involvement of Siglec-1 in NTHi phagocytosis by human alveolar macrophages, our data shed light on the importance of phagocytic receptors in alveolar macrophages in the pathogenesis of COPD.

## Supplementary information

**Additional file 1: Figure S1**. CD206 is a marker for alveolar macrophages in human lungs. A representative image of sorted FSC^hi^/SSC^hi^/CD45^+^/CD206^+^ cells by Diff-Quick stain. An error bar, 50 μm. Representative immunostaining for CD206 in lung tissues of control never-smokers (CNS, upper images), control ex-smokers (CES, middle images) and COPD ex-smokers (COPD, lower images). Right images are highly magnified images from each rectangle in left, corresponding images. Scale bars indicate 50 μm (left images), 25 μm (right images). Nuclei were counterstained with haematoxylin (blue). Histograms of CD14 expression on FSC^hi^/SSC^hi^/CD45^+^/CD206^+^ cells and on FSC^lo^/SSC^lo^/CD45^+^/CD206^−^ cells from human lung tissue lavages. FSC^hi^/SSC^hi^/CD45^+^/CD206^+^ cells showed little expression of CD14. FSC^lo^/SSC^lo^/CD45^+^/CD206^−^ cells contained CD14-highly expressing cells corresponding monocytes (an arrow) [1, 2]. Specific staining, red; isotype control, gray. **Fig. S2**. Siglec-1 expression in human alveolar macrophages. Representative immunostaining for Siglec-1 in lung tissues of five control never-smokers (a left image) and a negative control staining with only secondary antibody (a right image). Scale bars indicate 50 μm. **Table S1**. Antibodies for flow cytometry. **Table S2.** Isotype-matched control antibodies

## Data Availability

All of the datasets in the current study are available from the corresponding author on reasonable request.

## Abbreviations

BSA: Bovine serum albumin
CES: Control ex-smokers
CNS: Control never-smokers
COPD: Chronic obstructive pulmonary disease
CT: Computed tomography
eQTLs: Genome-wide expression quantitative trait loci
FcγRI: Fcγ receptor I
FEV1%pred: % predicted values of forced expiratory volume in 1 second
FMO: Fluorochrome minus one
GOLD: Global Initiative for Chronic Obstructive Lung Disease
IFN: Interferon
Ig: Immunoglobulin
ITAM: Immunoreceptor tyrosine-based activating motif
ITIM: Immunoreceptor tyrosine-based inhibitory motif
MARCO: Macrophage receptor with collagenous structure
MSR-1: macrophage scavenger receptor-1
NTHi: Non-typeable *Haemophilus influenzae*
PBS: Phosphate-buffered saline
ROS: Reactive oxygen species
SH2: Src homology 2
SNP: Single nucleotide polymorphism
Syk: Spleen tyrosine kinase
