# Supplementary material for: Decreased expression of a phagocytic receptor Siglec-1 on alveolar macrophages in chronic obstructive pulmonary disease
Source: Respir Res. 2020 Jan 28;21:30. doi: 10.1186/s12931-020-1297-2 (PMC6986024; doi:10.1186/s12931-020-1297-2)
Supplement: Supplementary file 1 — Additional file 1: Figure S1. CD206 is a marker for alveolar macrophages in human lungs. A representative image of sorted FSChi/SSChi/CD45+/CD206+ cells by Diff-Quick stain. An error bar, 50 μm. Representative immunostaining for CD206 in lung tissues of control never-smokers (CNS, upper images), control ex-smokers (CES, middle images) and COPD ex-smokers (COPD, lower images). Right images are highly magnified images from each rectangle in left, corresponding images. Scale bars indicate 50 μm (left images), 25 μm (right images). Nuclei were counterstained with haematoxylin (blue). Histograms of CD14 expression on FSChi/SSChi/CD45+/CD206+ cells and on FSClo/SSClo/CD45+/CD206− cells from human lung tissue lavages. FSChi/SSChi/CD45+/CD206+ cells showed little expression of CD14. FSClo/SSClo/CD45+/CD206− cells contained CD14-highly expressing cells corresponding monocytes (an arrow) [1, 2]. Specific staining, red; isotype control, gray. Fig. S2. Siglec-1 expression in human alveolar macrophages. Representative immunostaining for Siglec-1 in lung tissues of five control never-smokers (a left image) and a negative control staining with only secondary antibody (a right image). Scale bars indicate 50 μm. Table S1. Antibodies for flow cytometry. Table S2. Isotype-matched control antibodies [file 12931_2020_1297_MOESM1_ESM.docx]

*Online supplement*

**Decreased expression of a phagocytic receptor Siglec-1 on alveolar macrophages in chronic obstructive pulmonary disease**

**Author list:**

Atsushi Tanno^1†^, Naoya Fujino^1†*^, Mitsuhiro Yamada^1^, Hisatoshi Sugiura^1^, Taizou Hirano^1^, Rie Tanaka^1^, Hirohito Sano^1^, Satoshi Suzuki^2^, Yoshinori Okada^3^, Masakazu Ichinose^1^

**Supplementary Materials and Methods**

*Flow-cytometry analysis, fluorescence-activated cell sorting and Diff-Quik staining*

We collected lung hematopoietic cells including alveolar macrophages by perfusing and lavaging tissues with saline. The retrieved cell suspension was filtered through a 100 μm cell strainer (BD Biosciences, CA, USA) and treated with ACK lysing buffer (Thermo Fisher Scientific, MA, USA) for red blood cell lysis. After filtration with a 70 μm cell strainer (BD Biosciences), cells were re-suspended into 1% IgG/protease-free bovine serum albumin (BSA; Wako, Tokyo, Japan) in phosphate-buffered saline (PBS; Thermo Fisher Scientific).

Cell surface receptor expression was evaluated by flow cytometry with BD FACSCanto II (BD Biosciences). The cell suspension was stained with LIVE/DEAD Fixable Near-IR Dead Cell Stain Kit, for 633 or 635 nm excitation (Thermo Fisher Scientific). After treatment with an FcR blocking reagent (Miltenyi, Gladbach, Germany) which did not interfere with biding of an anti-FcγR1 antibody to Fcγ receptor, the cells were stained with fluorochrome-conjugated antibodies (Supplementary Table 1 and Supplementary Table 2). Controls were used as fluorochrome minus one (FMO) controls with corresponding isotype control antibodies. The expression intensity for phagocytic receptors was shown as a ratio of the geometric mean of fluorescence intensity for each antigen expression to the geometric mean of fluorescence intensity for the corresponding FMO with an isotype control. Data were analysed by FlowJo (Tree Star Inc., OR, USA).

An alveolar macrophage fraction defined as FSC^hi^/SSC^hi^/CD45^+^/CD206^+^ cells [1,2] was sorted using a FACS Aria II Cell Sorter and FACS Diva, version 6.1 (BD Biosciences). The sorted cells were cytospun and visualised with Diff-Quik Stain Kit (Sysmex, Kobe, Japan). Images were acquired on an OLYMPUS BX53 microscope using a 40x/ 0.75 UPlanFL N objective and captured using a DP71/u-TV1X-2 camera through a DP controller (Olympus, Tokyo, Japan).

*Immunohistochemistry for CD206 and Siglec-1on human lung sections*

Human resected lung tissues were fixed with 10% formalin overnight, embedded in paraffin and sectioned at a 3 μm thickness. Heat-induced antigen retrieval by autoclave (for 5 min at 121 °C) was performed using sodium citrate buffer (10 mM sodium citrate, 0.05% Tween20, pH 6.0) for CD206 or Tris-EDTA Buffer (10mM Tris Base, 1mM EDTA, 0.05% Tween 20, pH 9.0) for Siglec-1 staining. After blocking with 10% goat serum in PBS, sections were incubated with rabbit anti-human CD206 polyclonal antibody (Abcam, cat# ab64693, 1:1000) or rabbit anti-human Siglec-1 monoclonal antibody (Abcam, Cambridge, UK, cat# ab183356, clone SP216, 1:200) for overnight at 4 °C. After suppressing endogenous peroxidase activity, sections were incubated with a goat anti-rabbit antibody and horseradish peroxidase-conjugated polymer from a Histofine MAX-PO(R) kit as per the manufacturer's instructions (Nichirei, Tokyo, Japan). 3,3'-diaminobenzidine was applied for eight minutes to detect the antigen-antibody complex. Nuclei were counterstained with haematoxylin. Images were acquired on an OLYMPUS BX53 microscope using a 20x/ 0.50 or 40x/ 0.75 UPlanFL N objectives and captured using a DP71/u-TV1X-2 camera through a DP controller (Olympus).

**Supplementary Figure 1:** **CD206 is a marker for alveolar macrophages in human lungs.**

1. A representative image of sorted FSC^hi^/SSC^hi^/CD45^+^/CD206^+^ cells by Diff-Quick stain. An error bar, 50 μm.
2. Representative immunostaining for CD206 in lung tissues of control never-smokers (CNS, upper images), control ex-smokers (CES, middle images) and COPD ex-smokers (COPD, lower images). Right images are highly magnified images from each rectangle in left, corresponding images. Scale bars indicate 50 μm (left images), 25 μm (right images). Nuclei were counterstained with haematoxylin (blue).
3. Histograms of CD14 expression on FSC^hi^/SSC^hi^/CD45^+^/CD206^+^ cells and on FSC^lo^/SSC^lo^/CD45^+^/CD206^−^ cells from human lung tissue lavages. FSC^hi^/SSC^hi^/CD45^+^/CD206^+^ cells showed little expression of CD14. FSC^lo^/SSC^lo^/CD45^+^/CD206^−^ cells contained CD14-highly expressing cells corresponding monocytes (an arrow) [1,2]. Specific staining, red; isotype control, gray.

**
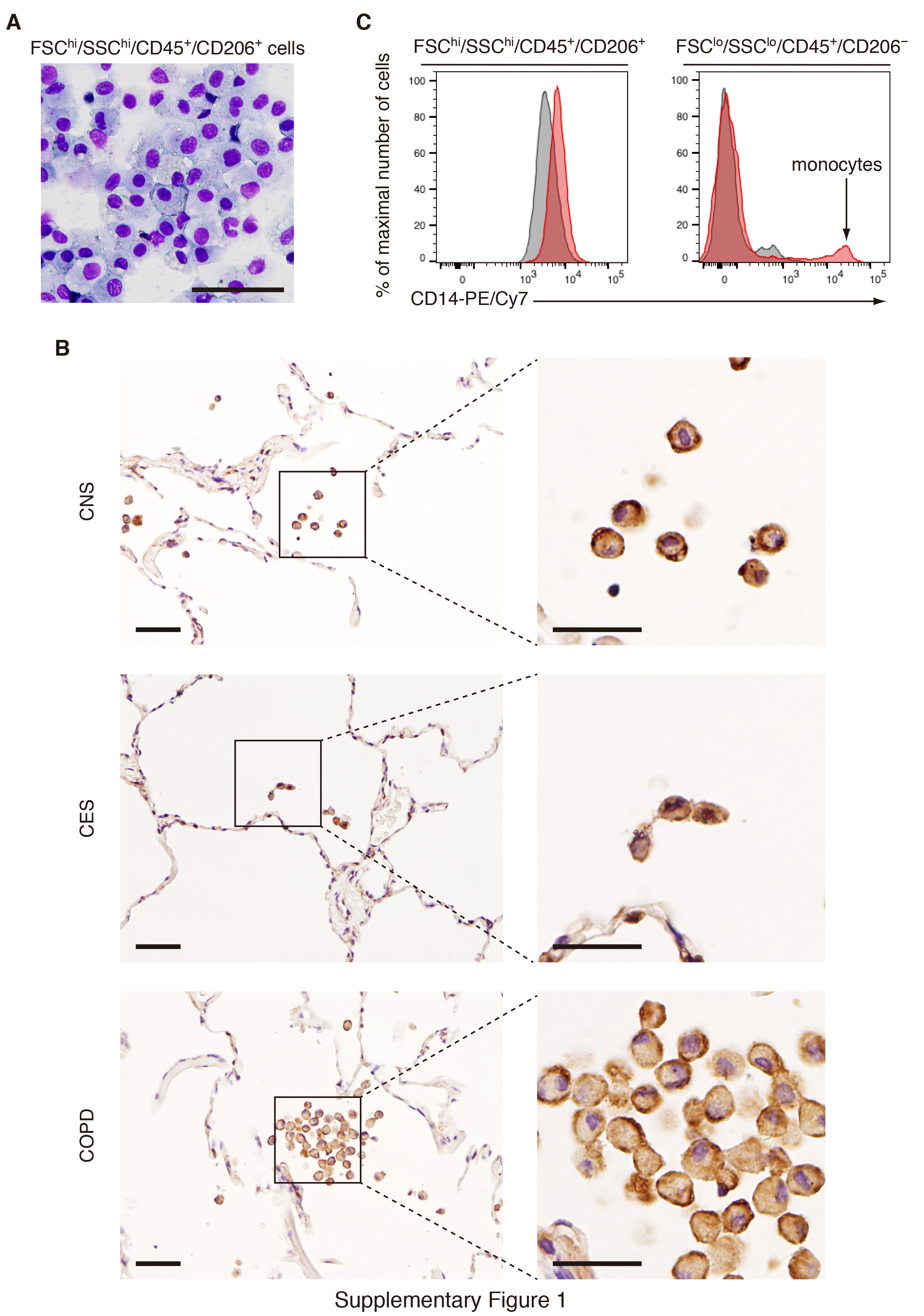
**

**Supplementary Figure 2: Siglec-1 expression in human alveolar macrophages.**


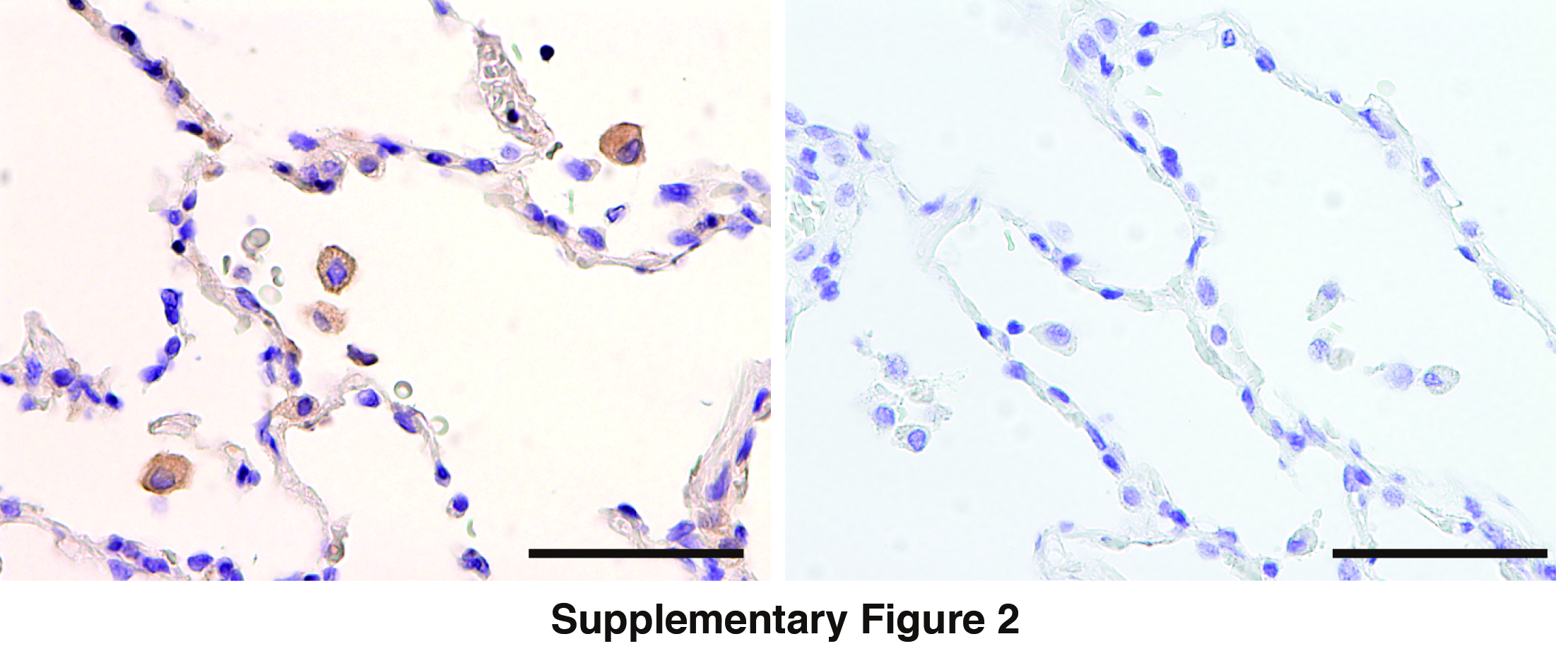
Representative immunostaining for Siglec-1 in lung tissues of five control never-smokers (a left image) and a negative control staining with only secondary antibody (a right image). Scale bars indicate 50 μm.

**Supplementary Table 1: Antibodies for flow cytometry**

| **Antigen** | **Fluorochrome** | **Vendor** | **Cat#** | **Isotype** | **Clone** | **W.C./D.F.** |
| --- | --- | --- | --- | --- | --- | --- |
| CD45 | BV421 | Biolegend | 304032 | Ms IgG1k | HI30 | 0.25 μg/mL |
| CD206 | APC | eBioscience | 17-2069-42 | Ms IgG1k | 19.2 | 0.24 μg/mL |
| FcγRI | PE | Biolegend | 305008 | Ms IgG1k | 10.1 | 2 μg/mL |
| CD11b | PE | Biolegend | 301306 | Ms IgG1k | ICRF44 | 1.5 μg/mL |
| MSR-1 | PE | R&D | FAB2708P | Ms IgG2b | 351615 | 10-fold dilution |
| CD36 | PE | Biolegend | 336206 | Ms IgG2a | 5-271 | 2 μg/mL |
| Siglec-1 | PE | eBioscience | 12-1699-42 | Ms IgG1 | 7-239 | 2 μg/mL |

W.C., working concentration; D.F., dilution factor; BV, Brilliant Violet; Ms, mouse; APC, allophycocyanin; PE, phycoerythrin

**Supplementary Table 2: Isotype-matched control antibodies**

| **Isotype controls-Fluorochrome** | **Vendor** | **Cat#** | **Working concentration** | **Indication** |
| --- | --- | --- | --- | --- |
| Mouse IgG1k-APC | eBioscience | 12-4714-41 | 2 μg/mL | for FcγRI, CD11b, Siglec-1 |
| Mouse IgG2a-PE | Biolegend | 400214 | 2 μg/mL | for CD36 |
| Mouse IgG2b-PE | R&D | IC0041P | 10-fold dilution | for MSR-1 |
| Mouse IgG1k-PE | eBioscience | 17-4714-41 | 0.24 μg/mL | for CD206 |

APC, allophycocyanin; PE, phycoerythrin

**References**

1. Yu Y-RA, Hotten DF, Malakhau Y, Volker E, Ghio AJ, Noble PW, et al. Flow Cytometric Analysis of Myeloid Cells in Human Blood, Bronchoalveolar Lavage, and Lung Tissues. Am J Respir Cell Mol Biol. 2016;54:13–24.

2. Bharat A, Bhorade SM, Morales-Nebreda L, McQuattie-Pimentel AC, Soberanes S, Ridge K, et al. Flow Cytometry Reveals Similarities Between Lung Macrophages in Humans and Mice. Am J Respir Cell Mol Biol. 2016;54:147–9.
